# Bayesian model with application to a study of dental caries

**DOI:** 10.1186/s12903-018-0687-z

**Published:** 2019-01-07

**Authors:** Mekuanint Simeneh Workie, Denekew Bitew Belay

**Affiliations:** 1 Mathematical and Statistical Modeling (Statistics), Bahir Dar University Institute of Technology, Bahir Dar, Ethiopia; 2 Department of Statistics, Hawassa University, Hawassa, Ethiopia

**Keywords:** Bayesian approach, Dental caries, Binary logistic regression, MCMC, Posterior distribution, Prior density

## Abstract

**Background:**

Dental caries are a significant public health problem. It is a disease with multifactorial causes. In Sub-Sahara Africa, Ethiopia is one of the countries with a high record of dental caries. This study was to determine the risk factors affecting dental caries using both Bayesian and classical approaches.

**Methods:**

The study design was a retrospective cohort study in the period of March 2009 to March 2013 dental caries patients Hawassa Haik Poly Higher Clinic. The Bayesian logistic regression procedure was adapted to make inference about the parameters of a logistic regression model. The purpose of this method was generating the posterior distribution of the unknown parameters given both the data and some prior density for the unknown parameters.

**Results:**

From this study the prevalence of natural dental caries was 87% and non-natural dental caries were 13%. The age group of 18–25 was higher prevalence of dental caries than the other age groups. From Bayesian logistic regression, we found out that rural patients, do not clean their teeth, patients from SNNPR and age group 18–25 are statistically significant. The finding from the Bayesian statistics approach is getting popular in data analysis than classical statistics because the technique is more robust and precise.

**Conclusions:**

Bayesian approach was found to be better than classical method as the value of the standard errors in Bayesian approaches is smaller than that of classical logistic regression. The Bayesian credible interval is smaller than the length of the confidence interval for all significant risk factors. Age, sex, place of residence, region and habit of cleaning teeth was found to have a significant effect on dental caries patients.

## Background

Dental caries is a microbial, multifactorial disease that succeeds in destroying the hardest substance of the human body, the enamel [1]. This disease is identified by the World Health Organization (WHO) as one of the most important public health issues [2]. Now a day dental caries on the rise to become major public health problems worldwide, nearly 60–90% of children and about 100% of adults have dental cavities, often leading to pain and discomfort [3].

The problem related with dental caries leads to a decrease in the quality of life of the affected individuals and society, with disparities related to well-known issues of socioeconomic, lack of preventive efforts, and dietary changes [4]. The burden of dental caries can affect school attendance, eating and speaking which leads to impair growth and development [5, 6].

Dental caries is one of the public health problems in both developed and developing countries [7]. Deteriorating oral health is an emerging public health concern in developing countries, yet little attention has been given to oral health in most sub-Saharan countries. The extents of caries, periodontal diseases and the associated risk factors have not been widely studied at the community level [8]. It is increasing gradually due to the growing consumption of sugary substances and poor oral care practices and inadequate health service utilization [9]. Ethiopia previous studies showed that, there were differences in different localities with regard to the prevalence of dental caries; 48.5% in Finote Selam, Ethiopia [10], 21.8% in Bahir dar city Ethiopia [11] and 78.2% in Debre Tabor General Hospital dental clinic [11, 12].

Dental caries causes tooth pain, discomfort, eating impairment, loss of tooth and delay language development. Furthermore, dental caries has effects on children’s concentration in school and a financial burden on the families [13, 14]. Risk factors such as sex, age, dietary habits, socioeconomic and oral hygiene status are associated with an increased prevalence and incidence of dental caries in a population [15]. The Person suffers from dental caries were examined for the type of dental caries in relation to different factors. The occurrence of dental caries was found to be slightly higher in females 51.45% [16].

Age is directly and strongly associated with prevalence of dental caries with increasing age the number of surfaces affected by caries increases, plateauing at around 50 years of age [17]. Teeth should be cleaned thoroughly at least twice a day using a fluoride toothpaste. Brushing helps remove the plaque and food particles from the tooth surface and flossing helps remove the plaque and food particles from the areas between the teeth. In Ethiopia, existing dental health services are limited. Even though, dental caries are highest in the country, much is not known about the factors affecting it in the study area. Therefore, this study was to determine statistical association between dental caries and some risk factors among patients attending Dental Clinic in Hawssa Haik poly Higher Clinic.

## Methodology

The study design was a retrospective cohort study in the period of March 2009 to March 2013. Data were collected by reviewing the Dental caries patient cards and information sheets in the Hawssa Haik poly Higher Clinic. The study is only Dental caries patients who had under the treatment been followed up in the clinic. A total numbers of 6007 dental caries patients in the clinic were considered for this study. The dependent variable used in this study was dental care that is dichotomous as natural dental caries (y_i_ = 1) and non-natural dental caries (y_i_ = 0). The independent variables in this study are sex, age, region, place of residence and habit of cleaning of teeth. The statistical method used in this study is known as classical approach and Bayesian approach. Classical approach, logistic regression analysis is to find the best fitting model to describe the relationship between an outcome and risk factors where the outcome is dichotomous. It is used to investigate the effect of risk factors on the probability of having natural dental caries [18]. Logistic regression models use a logit link function and it is expressed as:

1 $$ \log\;it\left[{P}_i\right]={\beta}_o+{\beta}_1{X}_{1i}+{\beta}_2{X}_{2i}+.\dots +{\beta}_k{X}_{ki}. $$

Where P_i_ is the probability of experiencing the outcome of interest for subject i, and X_1i_,..., X_ki_ are risk factors and β_i_ denotes the i^th^ regression coefficient [19]. Based on this model, the effect of each risk factor on the outcome can be expressed as an odds ratio. Binary outcomes are common in retrospective studies such as cohort studies. Logistic regression yields an odds ratio that approximates the risk ratio when the risk outcomes is low (< 10%). A consensus has been reached in an extensive argument in much of the literature that the risk ratio is preferred over the odds ratio for retrospective studies in case of the risk outcome less than 10%.. To obtain a model-based estimate of risk ratios, log-binomial regression has been recommended. However, this model may fail to converge and many methods have been provided as an alternative in these situations as Robust Poisson [20]. Log-binomial regression model is similar to the logistic regression model, except that it assumes a log link instead of a logit link, hence providing risk ratios instead of odds ratios. It can be presented as,

2 $$ \log \left[{P}_i\right]={\beta}_o+{\beta}_1{X}_{1i}+{\beta}_2{X}_{2i}+.\dots +{\beta}_k{X}_{ki.} $$

Based on this model, the effect of each risk factor on the outcome can be expressed as a risk ratio. There may be challenges when using the log-binomial model to estimate the RR because when fitting the log-binomial model, especially given continuous variables, non-convergence may be an issue when the MLE is close to or on the boundary of the parameter space [21]. The log-binomial is commonly used to estimate the RR; the OR estimated using logistic regression is often used to approximate the RR when the outcome is rare. However, regardless of the prevalence of the outcome, logistic regression predicted exposed and unexposed risks may be used to estimate the RR. When maximum likelihood estimation is used to fit the logistic model, estimation of the standard error of the RR is difficult. To overcome such difficulty in the estimation of the SE of the RR and provide a flexible framework for modeling, we developed a Bayesian logistic regression (BLR) model to estimate the OR, with an associated credible interval.

The Bayesian modeling framework and current software for Bayesian analysis can meet these complex challenges in a straightforward manner. Thus, we extended the logistic regression model for estimating the parameters to the Bayesian frame work. In the Bayesian framework, there are three key components associated with parameter estimation: the prior distribution, the likelihood function, and the posterior distribution. Bayesian Inference starts with formulating a prior probability distribution over the unknown parameters β, which summarizes a set of beliefs of knowledge before we observations the data [22]. The likelihood function is expressed as:

3 $$ L\left(\beta |y\right)=\prod \limits_{i=1}^n{\left(\frac{e^{\beta_o+{\beta}_1{x}_1+.\dots {\beta}_p{x}_p}}{1+{e}^{\beta_o+{\beta}_1{x}_1+..\dots {\beta}_p{x}_p}}\right)}^{yi}{\left(1-\frac{e^{\beta_o+{\beta}_1{x}_1+.\dots {\beta}_p{x}_p}}{1+{e}^{\beta_o+{\beta}_1{x}_1+..\dots {\beta}_p{x}_p}}\right)}^{\left(1- yi\right)} $$

Where the dental caries for the subject i who has covariate vector x_i_, y_i_ indicates the natural dental caries (y_i_ = 1), or non - natural dental caries (y_i_ = 0) of the i^th^ subject. Prior distributions play a very important role in Bayesian statistics. We have no prior knowledge available for the parameters of the score vectors. As a result the choice of the prior distribution becomes a challenge. In this case we can use a non–informative prior on the parameters of the score- vectors. Results of the Bayesian non – informative logistic regression approach tend to mimic a Maximum Likelihood approach, but we must observe that this non–informative approach on parameters of the scores is not non–informative on the parameters of the original variables. For this study, the most common priors for logistic regression parameters, which has the form: β_j_ ∼ N(μ_j_, σ^2^_j_) was used. This implies the normal distribution with mean μ_j_ and with variance σ^2^_j_. It can be expressed as [4]:

4 $$ f\left({B}_j\right)=\frac{1}{\sqrt{2{\pi \sigma}_j^2}}\exp \left\{\frac{-1}{2}{\left(\frac{\beta_j-{\mu}_j}{\sigma_j}\right)}^2\right\}. $$

In the case of no available prior knowledge, we consider a normal distribution with mean μ_j_ = 0 and large variance. In this essay, we choose σ^2^_j_ = 1000. The posterior distribution is derived by multiplying the prior distributions of the parameters of the likelihood function given as follows:

5 $$ f\left(\beta /y\right)=\prod \limits_{i=1}^n\left[{\left(\frac{e^{\beta_o+{\beta}_1{x}_1+.\dots {\beta}_p{x}_p}}{1+{e}^{\beta_o+{\beta}_1{x}_1+..\dots {\beta}_p{x}_p}}\right)}^{yi}{\left(1-\frac{e^{\beta_o+{\beta}_1{x}_1+.\dots {\beta}_p{x}_p}}{1+{e}^{\beta_o+{\beta}_1{x}_1+..\dots {\beta}_p{x}_p}}\right)}^{1- yi}\right]\prod \limits_{j=0}^P\frac{1}{\sqrt{2{\pi \sigma}_j^2}}\exp \left\{\frac{-1}{2}{\left(\frac{\beta_j-{\mu}_j}{\sigma_j}\right)}^2\right\}. $$

This gives a complex posterior distribution that is complicated to converge to a known distribution. In order to determine the posterior distribution, we will use the MCMC in the simulation of the random numbers following the posterior distribution. The Markov chain Monte Carlo method is a general method that generates the estimates of β (unknown parameters) from appropriate distribution and then corrects the values generated to have a better estimate of the desired posterior distribution [23]. The Gibbs sampling algorithm is a method to generate an instance from the distribution of each variable in turn, conditional on the current values of the other variables. It is a special case of Metropolis-Hasting algorithm where the random value is always accepted. Suppose that we partition the parameter vectors of the interest into the components. The term convergence of an MCMC algorithm refers to whether the algorithm has reached its equilibrium (target) distribution [24]. Several diagnostic tests have been developed to monitor the convergence of the algorithm such as time series, Density, autocorrelation, Gelman Rubin [25].

### Results of analysis

In Table 1, the result shows that females have more natural dental caries than males. The age group of 18–25 was a higher prevalence of dental caries than the other age groups. The patients living in urban had natural dental caries higher risk than those who live in rural parts. For patients coming from the South Nation Nationality People Representative (SNNPR), the proposition of the natural dental caries was 80.4% and for those coming from other regions was 88.3%. About 87.4% of the patients who did not clean their teeth had natural dental caries and the remaining 12.6% had non- natural dental caries. The prevalence of the outcome of interest which is natural dental caries was 87% and remaining 13% of the patients are non- natural dental caries.

**Table 1:** Tabulation of the response variable with each explanatory variable

Variable	Categories	Dental caries	
		No -natural (%)	Natural (%)
Gender	Female	327 (11.6)	2501 (88.4)
	Male	451 (14.2)	2728 (85.8)
Residence	Urban	434 (11)	3510 (89)
	Rural	344 (16.7)	1719 (83.3)
Region	SNNPR	183 (19.6)	751 (80.4)
	Others	595 (11.7)	4478 (88.3)
Age	<=18	90 (9.4)	872 (90.6)
	18–25	295 (14.5)	1742 (85.5)
	26–35	211 (13.2)	1392 (86.8)
	> = 35	182 (13.0)	1223 (23.4)
Clean teeth	Yes	68 (18.2)	305 (81.8)
	No	710 (12.6)	4924 (87.4)

#### Time series plot

**Fig. 1:**
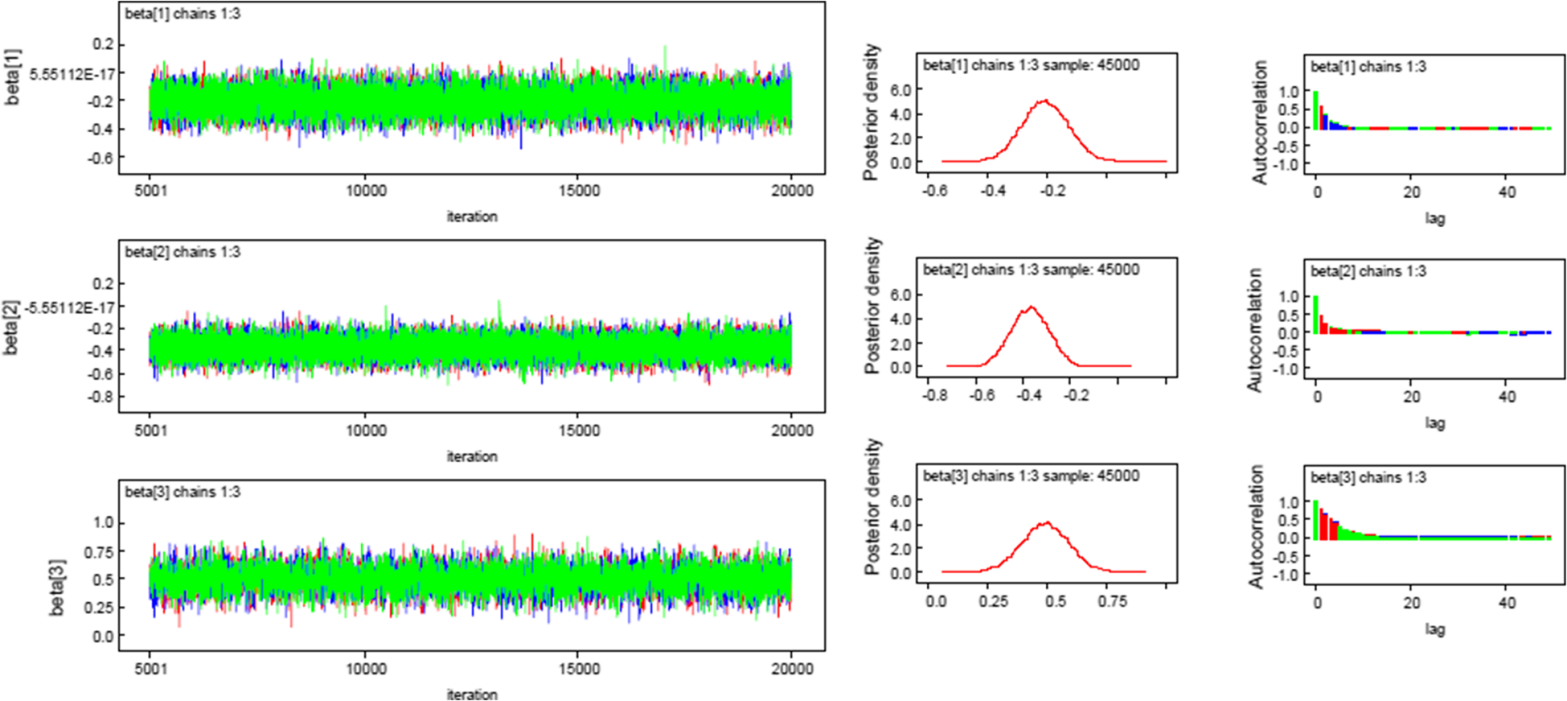
Convergence of Time Series, density and autocorrelation plots for the coefficients

**Fig. 2:**
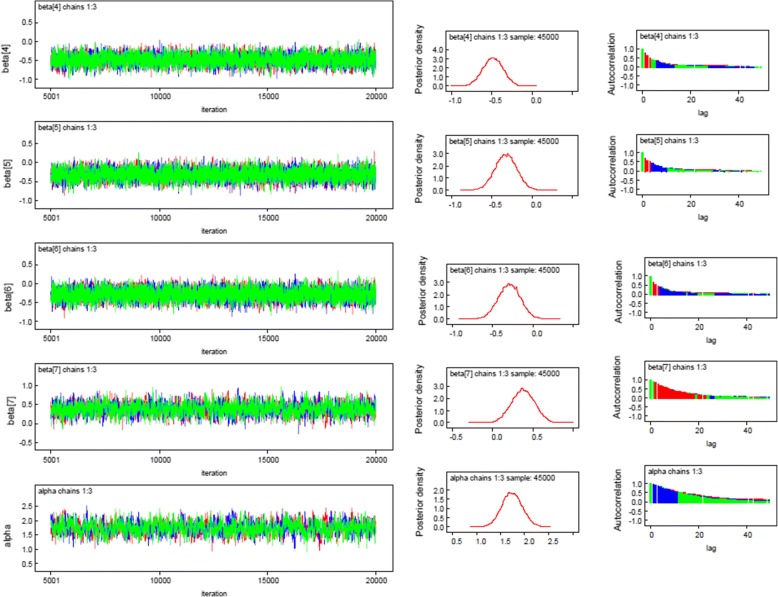
Time series, Density and Autocorrelation plot for convergence of coefficients for the predictors

It is one of the tests used to diagnosis the convergence of Bayesian analysis. Time series plot indicates a good convergence; three independent generated chains mixed together or overlapped (Fig. 1 and Appendix: Fig. 2).

#### Density plot

The plots for all risk factors indicate that the coefficient has bimodal density and hence the simulated parameter values were converged (Fig.1 and Appendix: Fig. 2).

#### Autocorrelation plot

From Fig. 1 and Appendix: Fig. 2, we observed that the autocorrelation for all parameters become low when we consider a lag equal to 50. Thus, an independent sample can be obtained by rerunning the algorithm with thin set equal to lag 50. The plots show that independent chains were mixed or overlapped to each other which confirm its convergences.

#### Gelman–Rubin statistics

It is one way of checking convergence in Bayesian analysis. It can be applied only when multiple chains are used. Gelman–Rubin convergence Statistics with the width of the pooled green, the average width of within the individual runs blue and their ratio for plotting purposes the pooled within the interval width are normalized to have an overall maximum of one (Appendix: Fig. 2).

### Results of classical approach

While the odds ratio (OR) is one of the most frequently used measures of association between a risk factor and an outcome in epidemiology, the risk ratio is important indices to quantify the strength of association between a given natural dental caries and a suspected risk factor. The main reason for the popularity of the OR is because the OR is the measure of association usually provided by logistic regression models. There is a large body of literature discussing the relationship between OR and RR. There is still an ongoing debate on the appropriateness of odds ratios versus prevalence ratios as measures of effect in retrospective cohort studies. It is known that the OR overestimates the RR when the outcome of interest is larger than 10%. The logistic model provided a better fit to the data relative to the log binomial and Poisson models, each of which can be problematic. Using a Poisson model with a robust standard error generally makes an adequate correction for the standard error. The log binomial model may fail to converge, which is not uncommon.

Table 2 show that, the odds ratio 0.617 which shows that the odds of natural dental caries are decreased by 38.3% for patients in the age group 18–25 compared to the patients in the age group ≤18 controlling for the other variables in the model. The odds of natural dental caries have decreased by 27.4% for patients with age group 25–35 compared to the reference group. The odds ratio 0.749 shows that the odds of natural dental caries have decreased by 25.1% for patients with age group > 35 compared to the reference category. The odds ratio 0.691 indicates that the odds of natural dental caries have decreased by 30.9% for patients in rural compared to those with urban controlling for the other variables in the model. The odds ratio 1.639 means that patients from SNNPR are 63.9% more likely to have natural dental caries than the patients from other regions controlling for other variables in the model.

**Table 2:** Model Summary for classical approach

Logistic					Robust Poisson		
Variables	Estimate(S.E.)	OR	95%CI	*p*-value	RR	95%CI	*p*-value
Intercept	1.723 (0.203)	5.600	1.3313, 2.1259	< 2e-16 ***	0.822	−0.350, − 0.043	4.705e-10 ***
Gender(ref = Female)							
Male	−0.2037 (0.0786)	0.8157	−0.3582,-0.0502	0.009502 **	0.974	−0.081, 0.028	0.007982 **
Residence (ref = urban)							
Rural	−0.3701 (0.0813)	0.6907	−0.5290,-0.2103	5.30e-06 ***	0.951	−0.110, 0.009	1.536e-05 ***
Region(ref = others)							
SNNPR	0.4943 (0.0970)	1.6393	0.3023,0.6827	3.47e-07 ***	1.081	−0.0012, 0.158	7.055e-06 ***
Age group(ref = < 18)							
18–25	− 0.48289 (0.12817)	0.6170	− 0.7387,-0.2357	0.000165 ***	0.945	−0.137, 0.0255	4.203e-05 ***
26–35	− 0.32040 (0.13420)	0.7259	− 0.5873,-0.0606	0.016969 *	0.966	−0.119, 0.051	0.015609 *
> =35	− 0.2884 (0.1376)	0.7494	− 0.56156,-0.0215	0.036091 *	0.970	− 0.117, 0.057	0.038829 *
Clean Teeth(ref = no)							
Yes	0.38855 (0.14164)	1.4748	0.10399,0.6600	0.006082 **	1.061	−0.0545, 0.177	0.017764 *

*p < 0.05

**p < 0.01

***p < 0.001

The OR = 1.475 indicates that patients clean their teeth were 47.5% more likely to have natural dental caries compared to patients did not clean their teeth controlling for the other variables in the model. The result gives an OR = 0.8157, this indicates that, male are 0.8157 less likely to have natural dental caries than female.

### Results of Bayesian approach

The finding in Table 3 show that, regarding the effects of gender on the dental caries, we found out that males are 81.6% most less likely to have natural dental caries compared to females. For patients who lived in urban have higher risk than those who lived in rural. Patients who lived in rural area have 69.1% less probable than those who live in urban area. The result also illustrated that patients from SNNPR are 64.2% more likely to be natural dental caries patient than other regions. Those who clean their teeth are 47.6% more likely to be natural dental caries than those who don’t clean their teeth. Dental caries patients in age group 18–25, age group 26–35 and age group > 35 have been 0.616, 0.726, 0.749 times less likely to be natural dental caries patient than in age group ≤18 respectively. The logistic and robust Poisson produced similar results with Bayesian logistic regression model. Bayesian logistic regression is a viable alternative to the log binomial and robust Poisson models to estimate the RR and associated CI.

**Table 3:** Model summary for Bayesian approach

Parameters	Mean(β)	S.E_β_	MC error	Median	HPD	
					2.5%	97.5%
α(intercept)	1.726	0.1976	0.003618	1.724	1.343	2.112
Gender(ref = Female)						
β1(Male)	−0.2038	0.07863	5.056E-4	−0.2039	− 0.3579	− 0.04931
Residence (ref = urban)						
β_2_ (Rural)	−0.3699	0.08083	5.049E-4	− 0.37	− 0.5282	− 0.2109
Region(ref = others)						
β_3_ (SNNPR)	0.4957	0.09676	9.845E-4	0.4963	0.3043	0.6835
Age group(ref = < 18)						
*β*_4_(18–25)	− 0.484	0.1279	0.001494	−0.4827	− 0.7383	− 0.2373
*β*_5_(26–35)	− 0.3205	0.1338	0.001507	− 0.3193	− 0.5867	− 0.06207
*β*_6_(> = 35)	− 0.2887	0.137	0.001513	−0.2879	− 0.5606	− 0.022
Clean Teeth(ref = no)						
*β*_7_(Yes)	0.389	0.1395	.002127	0.3909	0.1126	0.6593

### Model comparison

From Table 4, we made comparison of Bayesian and classical approaches and identified that more significant risk factors, numerical value differences in standard error. The important comparison method used is the standard error of the estimated parameters for both approaches. In Bayesian logistic regression approach all significant factors have smaller standard error than the classical logistic regression approach. From Table 4 results, we have found that the length of Bayesian credible is lower than the length of the confidence interval for all covariates in classical logistic regression. Therefore, we can say that the Bayesian approach provides better results using the confidence interval/credible interval and standard errors of the estimated parameters.

**Table 4:** The model comparison between classical approach and Bayesian approach

parameters	S.E. and Confidence interval for maximum likelihood estimators					SD and Credible interval for Bayesian estimator				
	Estimate	S.E.	Interval Estimate (95%)			β	Sd	Credible Interval (95%)		
			Lower	Upper	Length			Lower	upper	length
α(intercept)	1.723	0.203	1.3313	2.1259	0.795	1.7	0.1976	1.343	2.112	0.769
β1(Male)	−0.2037	0.078	−0.358	−0.050	0.308	−0.204	0.078	−0.3579	−0.04931	0.308
β_2_ (Rural)	−0.3701	0.0813	−0.5290	−0.2103	0.319	−0.3699	0.08083	−0.5282	− 0.2109	0.317
β_3_ (SNNPR)	0.4943	0.097	0.3023	0.6827	0.380	0.4957	0.09676	0.3043	0.6835	0.379
*β*_4_(18–25)	−0.4829	0.1282	−0.7387	− 0.2357	0.503	− 0.484	0.1279	− 0.7383	−0.2373	0.501
*β*_5_(26–35)	−0.3204	0.1342	−0.5873	−0.0606	0.527	−0.3205	0.1338	−0.5867	− 0.06207	0.5246
*β*_6_(> = 35)	−0.2884	0.138	−0.5616	−0.0215	0.540	−0.2887	0.137	−0.5606	− 0.02299	0.538
*β*_7_(Yes)	0.38855	0.142	0.10399	0.660	0.556	0.389	0.1395	0.1126	0.6593	0.5467

## Discussion

The prevalence of non-natural dental caries found in the present study was 13%. From this study we found that the odds ratio of being non-natural dental caries for males were higher than females. Similarly study done in Ethiopia about the prevalence of dental caries in North west Ethiopia showed the prevalence of dental caries was found to be different between male and female [26]. The highest proportion of dental caries is observed in the age group 18–25 on the other hand, the lowest proportion of dental caries in the age group < 18 which is supported by the study [9]. The urban patient is more likely to dental caries than rural patient. The reason could be patient who lives in urban areas tend to use more sweet consumption than rural patient. The paper [27] which shows that there are differences in oral health related behavior between urban and rural residences confirms our study. The prevalence of daily use of tooth picks was consistently and significantly higher among more urban than rural residence.

## Conclusions

In this study we tried to show the performance of Bayesian logistic regression over the classical logistic regression. The factors Age, gender, region, place of residence and habit of cleaning teeth were associated risk factors for dental caries. A comparison of the classical and Bayesian approach logistic regression reveals lower standard errors of the estimated coefficients in the Bayesian logistic regression approach. At the same time in Bayesian approach were used and compare with method of maximum likelihood and found that the length of the Bayesian credible interval is smaller than the length of the confidence interval for all factors.

## Figures and Tables

**Fig. 3:**
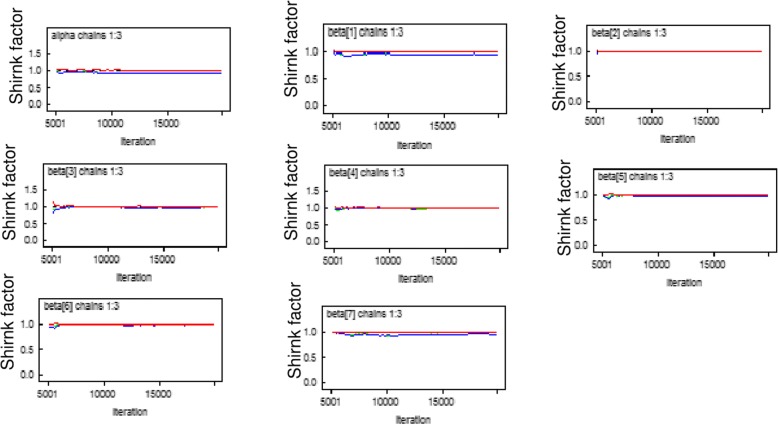
Gelman–Rubin statistic plot for convergence of coefficients for the predictors

## Abbreviations

CI: Confidence Interval
HPD: Highest Posterior Density
MCMC: Markov chain Monte Carlo
OR: Odds Ratio
SNNPR: South Nation Nationality People
winBUGS: Win Bayesian using Gibbs sampling
